# Cross-Sectional Study to Establish the Utility of Serum Tumor Markers in the Diagnosis of Lung Cancer

**DOI:** 10.31557/APJCP.2021.22.8.2569

**Published:** 2021-08

**Authors:** Anurag Mehta, Anuj Parkash, Meenu Bhatia

**Affiliations:** *Rajiv Gandhi Cancer Institute and Research Centre. India. *

**Keywords:** Lung cancer, tumour markers, CEA- CYFRA 21-1, ProGRP, NSE, SCC

## Abstract

**Background:**

Reliable blood markers for aiding lung cancer (LC) diagnosis and differentiating LC from tuberculosis are lacking in India.

**Methodology:**

In this single-centre, cross-sectional, real-world study, serum levels of 5 TMs (CEA, CYFRA 21-1, SCC, ProGRP, NSE) were measured from consented patients with suspicious lung nodules who were candidates for biopsy, and also from healthy controls. TM level measurement was done through electrochemiluminescent immunoassay, followed by histological diagnosis on the biopsied specimen. Using package insert cut-offs, sensitivity and specificity of the 5 TMs were evaluated individually and in combination. Using receiver operating characteristic (ROC) curves of individual TMs, the ability of CEA, CYFRA 21-1, and ProGRP taken together was evaluated for its ability to differentiate LC from no-LC.

**Results:**

Out of 178 subjects, 160 had LC (147 NSCLC; 13 SCLC). NSCLC patients had higher median values of CYFRA 21-1 and SCC; SCLC patients had higher median values of CEA, NSE, and ProGRP. Adenocarcinoma-NSCLC patients had higher median values of CEA, CYFRA 21-1, NSE, and ProGRP; squamous-NSCLC patients had higher median value of SCC. For differentiating LC from no-LC, the combination of all 5 TMs (sensitivity:97.5%, specificity:33.3%) and combination of CEA, CYFRA 21-1 and ProGRP (sensitivity:91.3%, specificity:88.9%) were found suitable.

**Conclusion:**

Combination of all 5 TMs, and combination of CEA, CYFRA 21-1, and ProGRP represents an easy and non-invasive method for aid in LC diagnosis that does not require technical expertise. TM evaluation can also supplement histological diagnosis of LC.

## Introduction

As per the Globocan 2020 data, lung cancer (LC) accounted for 11.4% of all new cases of cancer worldwide, second only to breast cancer, and 18% of all global cancer-related mortality was due to LC (WHO-IARC, 2020a). It is estimated that the global number of patients with cancer involving trachea, bronchus, and lung will increase from 2.21 million in 2020 to 3.63 million in 2040 (WHO-IARC, 2020d). LC is a significant public health problem in India, with an estimated 72,510 new cases of LC, and 66,279 deaths due to LC annually (WHO-IARC, 2020b). By 2040, it is predicted that in India there will be 120,000 new cases, and 110,000 deaths attributable to, cancers involving trachea, bronchus, and lung (WHO-IARC, 2020c). The most common cause of LC is mostly attributable to smoking, including passive smoking, which is responsible for at least 90% of all cases of LC (Siddiqui and Siddiqui, 2020). The two most frequent histological varieties of LC include Non-small cell LC (NSCLC, around 85% of all LCs) and small cell LC (SCLC, around 15% of all LCs). NSCLC is further classified into three histological subtypes: adenocarcinoma, squamous cell carcinoma, and NSCLC not otherwise specified (NOS) (Inamura, 2017; Dang et al., 2020; Siddiqui and Siddiqui, 2020). Though biopsy is necessary to establish the diagnosis of LC, it is invasive, and risk-prone (Liu et al., 2017). Pre-selection of patients with highly sensitive tumour biomarkers can reduce the number of patients undergoing biopsy. 

LC is associated with a high mortality rate: as per the American Lung Association, the 5-year survival rate of LC is 56% for localised cancer, but as low as 5% for metastatic cancer (ALA, 2020). Factors influencing this delay in LC diagnosis include asymptomatic nature of early disease or non-specific symptoms such as dyspnoea, cough, loss of weight, loss of appetite, and shortness of breath (Xing et al., 2019). Another peculiar factor hindering the early diagnosis of LC in India is the high local prevalence of tuberculosis, which has many overlapping clinical features (such as anorexia, weight loss, hemoptysis, cough, and fever) and radiological features with LC (Çakar and Çiledağ, 2018). As a result, patients with LC are often misdiagnosed as pulmonary tuberculosis, resulting in a delay in the correct diagnosis and appropriate treatment, and lower survival (Bhatt et al., 2012; Parker et al., 2018).Cancer screening techniques with high sensitivity can aid in the diagnosis of LC by selection of cases likely to have cancer so that they can be referred to tertiary care centre for appropriate further evaluation and management immediately.

The journey of cancer patients in India usually starts from a non-specialist general hospital, which in many cases are the government-run primary health centres (PHCs) or community health centres (CHCs), that largely cater to the more common illnesses. In the backdrop of inadequate resources at these centres, coupled with the generally poor healthcare access at these peripheral health centres, and lack of specialist facility for cancer diagnosis, it may take a considerable time for even a suspicion or a provisional diagnosis of cancer in a patient, and subsequent referral to a speciality cancer care centre. Such a delay may prove fatal especially with LC, which is already associated with a high mortality rate (Sirohi, 2014; Dang et al., 2020; Dang et al., 2021). Thus, efforts should be made to identify avenues that help primary care physicians (PCPs) to arrive at a provisional diagnosis of LC in the peripheral centres, enabling prompt referral, early diagnosis and subsequent early initiation of treatment for LC.

Serum tumour markers (TMs) can solve all the three problems identified above (Molina et al., 2016). They are non-invasive and convenient options that can complement histological diagnosis of the biopsied specimen, they can be interpreted by a PCP without much difficulty, and can be used to aid the diagnosis of LC in patients with overlapping symptoms of tuberculosis and LC at the PHC/ CHC level. Abnormal TM levels can be used as basis for referral of patients with suspected LC to higher centres for further evaluation. However, data on the utility of these TMs for LC diagnosis among Indian patients is scarce. With this background, we were interested to evaluate the role of five TMs –carcinoembryonic antigen (CEA), cytokeratin fragment 21-1 (CYFRA 21-1), squamous cell carcinoma–associated antigen (SCC), pro–gastrin-releasing peptide (ProGRP), and neuron-speciﬁc enolase (NSE)– in the diagnosis of LC. We were interested in these five specific biomarkers since previous studies have suggested a correlation of histological tumour types with TMs: adenocarcinoma with CEA; squamous cell carcinoma with CYFRA 21-1 and SCC; large cell carcinoma with CYFRA 21-1 and NSE; and small cell lung cancer with NSE and ProGRP (Molina et al., 2010). The specific objective of this study was to determine the utility of these 5 serum tumor markers – either alone or in combination – in differentiating LC from benign lesions, and in identifying the histological type of LC.

## Materials and Methods

*Study site, Subjects, and Ethics Committee Approval*

This single centre, diagnostic utility, open label, cross-sectional study was conducted in a real-life setting, after obtaining institutional ethics committee approval to the study protocol. Adult patients of either sex referred to the Department of Oncology at Rajiv Gandhi Cancer Institute and Research Centre, New Delhi, were screened for inclusion in the study. Patients with suspicious lung opacities in whom tissue biopsy was indicated based on the radiological findings such as ground glass or solid/ spiculated opacity were briefly explained about the study, and consenting patients were recruited into the study after obtaining their signature on the informed consent form. In addition, consenting healthy volunteers with no pulmonological symptoms suggestive of either tuberculosis or LC were recruited to serve as healthy controls. We included newly diagnosed cases of LC of all tumour stages, who were oncology treatment-naïve, and excluded patients who did not have a successful biopsy, patients not giving consent to participate in the study, and patients with known liver or kidney disease. The study was conducted in accordance with the ethical standards as enshrined in the Declaration of Helsinki and all other applicable ethical documents, and consent for publication was obtained from all recruited patients. Upon patient recruitment into the study, baseline and demographic data was collected. Data pertaining to the presenting symptoms, smoking history, and details of the pulmonary nodule were documented in a predetermined data extraction grid. For the purpose of this study, patients with passive smoking were not considered separately and clubbed together with those who gave active smoking history, and patients giving history of Gutka chewing or tobacco chewing (and not smoking) were considered as not having history of smoking.

*Serum TM levels*

Blood samples were obtained through peripheral venepuncture, and serum levels of CEA, CYFRA 21-1, SCC, ProGRP, and NSE were measured by using a commercially available electro-chemiluminescent immunoassay (ECLIA) test kits from Roche diagnostics through the cobas e411 equipment (Roche Diagnostics, Mannheim, Germany) coupled with the Roche Elecsys® Platform (Roche Diagnostics, Manheim, Germany). The samples were collected using standard sampling tubes containing serum separating gel and centrifuged at 3000 RPM for 15 minutes and serum was separated. The serum of ProGRP, SCC, CYFRA 21-1 and CEA was stored in aliquots at -20^o^C and serum of NSE was first deep freeze at -70^o^C and then stored at. -20^o^C. At the time of analysis, the frozen samples were thawed one time, at ambient temperature and measured within 2 hours. 

As per the package inserts for the assays, the upper limits considered in the present study for the 5 TMs were: 4.7 ng/ml for CEA; 3.3 ng/ml for CYFRA 21-1; 2.6 ng/ml for SCC;68.3 pg/ml for ProGRP; and 16.3 ng/ml for NSE.(2013; 2016a; 2016d; 2016c; 2016b) Any individual TM value above these cut-offs were considered to be abnormal. When evaluating TMs in combination, presence of at least one abnormal TM value among the selected combination was considered to be abnormal test result. The package insert cut-offs were used to calculate sensitivity and specificity for differentiating LC cases and non-LC controls, and also for differentiating NSCLC and SCLC among the LC cases. Using the sensitivity and specificity values, we constructed receiver operating characteristic (ROC) curves, and the resulting area under the curves (AUCs) were used for determining the most appropriate combinations of TMs to be used for distinguishing LC from no LC, and for distinguishing NSCLC from SCLC. Subsequently, using the TM levels obtained from the participants in this study, study-specific TM cut-off values were developed by drawing ROC curves and identifying the TM levels with the best values for sensitivity and specificity; these cut-offs were further evaluated for their ability to differentiate LC from no LC, and NSCLC from SCLC. 

*Biopsy and Histological Classification*

After obtaining the serum samples, the biopsied sample of the suspicious lesion was processed through Haemotoxylin and Eosin staining and immunohistochemistry, following the hospital protocol. Clinical or pathological staging was based on AJCC 7^th^ edition guidelines, (Rusch et al., 2009) and the diagnosis of LC was done per the 2015 World Health Organization recommendations (Travis et al., 2015). The biopsy was conducted as part of the patient’s routine investigation and not as a separate procedure for the study. Biopsy was not performed on healthy volunteers.

*Sample size*

Considering the pre-determined values of sensitivity of ProGRP <50 pg/ml as 17.1%, with 5% margin of error and 95% confidence level, the sample size was determined to be 218 patients. Since the sensitivities of other evaluated biomarkers were expected to be greater than ProGRP, sensitivity of ProGRP was used for sample size calculation. 

*Data Management and Statistics*

All data were entered in Microsoft Excel 2016; statistical analysis was performed using SPSS version 20.0 (IBM Corp. Released 2011. IBM SPSS Statistics for Windows, Version 20.0. Armonk, NY: IBM Corp) and MedCalc Statistical Software version 19.2.6 (MedCalc Software Ltd, Ostend, Belgium; https://www.medcalc.org; 2020. Results were expressed as number, proportion, mean, median, and interquartile range (IQR) based on the nature of the variable. Standard formulae were applied to derive sensitivity and specificity. Non-parametric tests (Mann Whitney U test and Kruskal Wallis Test) were used to compare TM levels, parametric tests (t-test, ANOVA) were used to compare age and nodule size, and Pearson chi-square test was used to compare proportions between groups. DeLong model was used to construct ROC curves and compare the AUCs. For all the tests, p value of <0.05 was considered to be significant.

*Data Availability*

All datasets leading to the results of the study are available with the corresponding author on reasonable request.

## Results

*Patient recruitment and baseline characteristics*

Patient recruitment started in May 2018. Even though it was planned to recruit 218 patients for this study, due to the onset of the COVID-19 pandemic and the associated lockdown and related restrictions starting from March 2019, we found it difficult to recruit the targeted number of patients. To respect timelines, we ended patient recruitment in May 2020, and by this time we were able to recruit 178 subjects, 40 short of our target.

Out of the 178 recruited subjects, a diagnosis of LC was confirmed in 160 patients (89.89%). NSCLC and SCLC were found in 147 and 13 patients respectively. Among NSCLC patients, adenocarcinoma, squamous carcinoma, and large cell carcinoma was found in 96, 38, and 1 patient respectively, with 12 patients receiving a diagnosis of NSCLC-NOS. Among the 18 subjects without LC (10.11%), 5 were healthy volunteers, 10 patients had tuberculosis, and one patient each had a diagnosis of fibromatosis, histoplasmosis, and lung abscess. The three most frequent presenting complaint of the subjects were persistent cough (38.8%), hemoptysis (12.9%), and breathing difficulty (10.7%). All presenting complaints were more frequent in cases compared to controls. 

*Comparison between different groups of patients*

Table 1 summarises the comparison of various baseline parameters between different patient groups. Subjects with LC were older, and had more frequent history of smoking than those without LC. Among the 160 patients with LC, age distribution, gender distribution, and smoking history were comparable between patients with NSCLC and SCLC. Among the 147 patients with NSCLC, the age distribution was comparable between patients with adenocarcinoma and squamous NSCLC. Proportion of males was higher, and smoking history was more often seen in patients with squamous NSCLC compared to adenocarcinoma.

*LC vs No LC*

**Figure 1:**
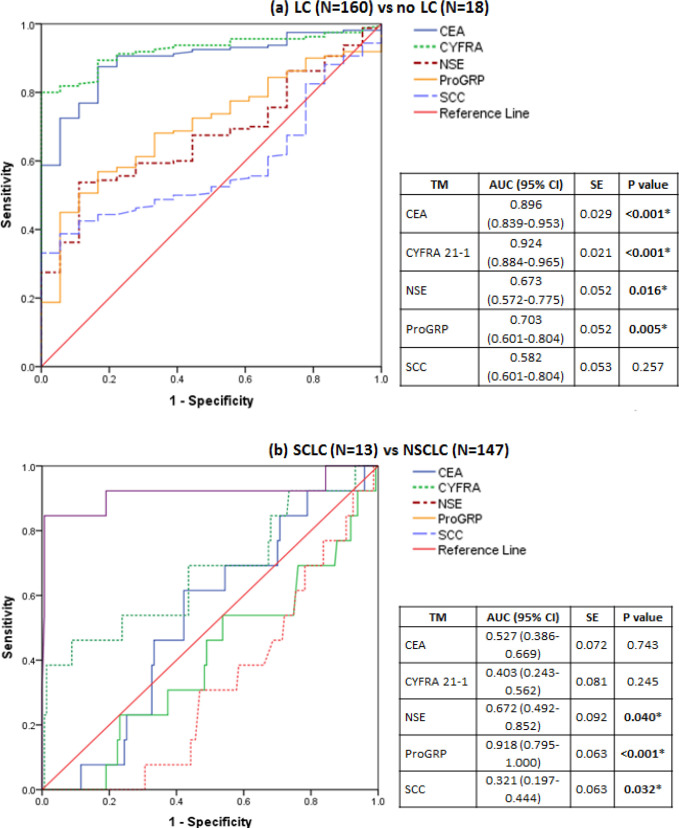
Receiver Operating Characteristic Curves of Individual TMs to Differentiate between (a) lung cancer cases and non-lung cancer controls; (b) SCLC from NSCLC. Cut-offs given in the package inserts of the individual TMs were used to derive these curves. Note: TM, tumour markers; AUC, area under the curve; LC, lung cancer; NSCLC, non-small cell lung cancer; SCLC, small cell lung cancer; CEA, carcinoembryonic antigen; CYFRA 21-1, cytokeratin fragment 21-1; SCC, squamous cell carcinoma–associated antigen; ProGRP, pro–gastrin-releasing peptide; NSE, neuron-speciﬁc enolase; *, statistically significant finding

Median values of all 5 TMs were higher among patients with LC compared to no LC; comparisons of CEA, CYFRA 21-1, NSE, and ProGRP reached statistical significance (Table 2). However, with the no LC group having a disproportionately small size, the statistical significance observed here must be interpreted with caution. For differentiating between LC and no LC, when the TMs were considered individually, CEA, CYFRA 21-1, ProGRP, and SCC had high specificity but low sensitivity. When all the 5 TMs were considered together, the sensitivity of detection of a LC case when any one of the 5 TMs was having abnormal value, was very high at 97.5%, indicating that the combination of 5 TMs has a high value for screening for LC cases; however, the specificity of this combination was 33.3% indicating a low true positive rate. (Table 3, Figure 1a) As before, this observation is to be interpreted in the context of low numbers of patients with no LC.

Based on the AUCs observed in the ROC curves, we further explored the diagnostic capability of using TMs in combination. The combination of CEA, CYFRA 21-1, and ProGRP was found to have high values for sensitivity (91.3%) and specificity (88.9%) indicating that this combination has an optimal capability to aid in LC diagnosis (Table 3).

*NSCLC vs SCLC*

When TM levels in patients with NSCLC and SCLC were compared, median values of CYFRA 21-1 and SCC were higher among NSCLC patients, and median values of CEA, NSE, and ProGRP were higher among SCLC patients; comparisons of NSE, ProGRP, and SCC reached statistical significance. This finding should be interpreted in the context of low number of patients with SCLC. When TM levels in patients with adenocarcinoma and squamous variants of NSCLC were compared, the median values of CEA, CYFRA 21-1, NSE, and ProGRP were higher among patients with adenocarcinoma, and median value of SCC was higher among patients with squamous NSCLC; statistical significance was found with CEA, NSE, and SCC (Table 2). For identifying patients with NSCLC from those with SCLC, ProGRP had the best values of sensitivity (92.3%) and specificity (74.1%) among the 5 TMs considered (Table 3, Figure 1b).

*Deriving study-specific TM cut-off values*

Based on the TM levels observed in this study, we derived the study-specific TM cut-off values which has the best combination of sensitivity and specificity. Subsequently, we also evaluated the combination of TMs based on these study-specific cut-offs for their ability to distinguish cases from controls (Table 4). An adjustment of cut-off levels of the different TMs could further optimize overall performance for aid in LC diagnosis with better values of sensitivity and specificity, as compared to using package insert cut-offs. When all the 5 TMs were considered together, the sensitivity of LC diagnosis was 99.4%, and specificity was 50.0%, with the adjusted cut-offs. Further, like before, the combination of CEA, CYFRA 21-1, and ProGRP showed excellent malignancy detection capability, with a sensitivity of 97.5% and a specificity of 72.2% for identifying LC patients. The sensitivity and specificity values of both the combinations were higher with the adjusted cut-offs compared to the package insert cut-offs.

**Table 1:** Comparison of Baseline Features between Different Patient Groups

Patient group	Overall (N=178)	LC (N=160)	No LC (N=18)	NSCLC (N=147)	SCLC (N=13)	Adenocarcinoma NSCLC (N=96)	Squamous NSCLC (N=38)	Other variants of NSCLC (N=13)
Age, yrs (Mean ± SD)	59.52 ± 12.97	60.74 ± 11.35	48.72 ± 20.21	61.13 ± 10.96	56.31 ± 14.86	60.24 ± 11.27	61.89 ± 10.68	65.46 ± 8.77
Males (N, %)	125 (70.22%)	112 (70%)	13 (72.22%)	100 (68.03%)	12 (92.31%)	57 (59.38%)	33 (86.84%)	10 (76.92%)
Smoking History (N, %)	82 (46.1%)	82 (100%)	0 (0%)	73 (49.66%)	9 (69.23%)	38 (39.58%)	25 (65.79%)	10 (76.92%)
Presence of nodule (N, %)	135 (75.8%)	132 (97.8%)	3 (2.2%)	121 (82.31%)	11 (84.62%)	82 (85.42%)	28 (73.68%)	11 (84.62%)
Nodule size, cm	4.5	4.45	4.5	4.5	4.4	3.85	5	7.5
(Median-IQR)	(2.90-6.50)	(2.90-6.50)	(2.50-NA)	(2.95-6.50)	(2.90-7.30)	(2.88-5.58)	(2.83-6.65)	(5.95-7.95)

IQR, interquartile range; LC, lung cancer; NSCLC, non-small cell lung cancer; SCLC, small cell lung cancer; Other variants, 1 patient had large cell carcinoma, and 12 patients had NSCLC-NOS

**Table 2:** Comparison of Median (Interquartile range) TM Levels between Different Patient Groups

Tumor marker	Cut off	LC (N=160)	No LC (N=18)	"P value (LC vs no LC)"	NSCLC (N=147)	SCLC (N=13)	"P value (NSCLC vs SCLC)"	Adeno carcinoma (N=96)	Squamous NSCLC (N=38)	"P value (adeno vs squamous)"
CEA (ng/ml)	4.7	8.38 (2.95-34.36)	1.68 (0.93-2.09)	<0.001*	8.21 (2.92-34.52)	10.09 (3.48-26.13)	0.743	13.02 (4.68-54.55)	3.40 (2.11-6.26)	<0.001*
CYFRA 21-1 (ng/ml)	3.3	7.31 (3.63-17.72)	1.75 (1.37-2.17)	<0.001*	7.44 (3.66-17.89)	6.76 (2.36-15.83)	0.245	9.61 (3.89-24.68)	5.65 (2.64-13.00)	0.075
SCC (ng/ml)	2.6	1.93 (1.18-4.94)	1.85 (1.28-2.21)	0.257	2.02 (1.23-5.48)	1.29 (0.89-2.23)	0.032*	1.52 (1.00-3.11)	5.69 (2.19-11.38)	<0.001*
ProGRP (pg/ml)	68.3	52.99 (37.63-75.3)	38.80 (26.93-49.75)	0.005*	51.39 (35.62-69.59)	5000.00 (924.70-6000.00)	<0.001*	51.13 (34.39-69.42)	49.85 (35.19-62.66)	0.682
NSE (ng/ml)	16.3	21.55 (13.44-38.48)	16.50 (9.96-19.54)	0.016*	21.11 (13.23-36.28)	36.69 (16.21-185.10)	0.04*	21.38 (14.10-39.95)	16.09 (11.41-25.89)	0.034*

Note: TM, tumour marker; LC, lung cancer; NSCLC, non-small cell lung cancer; SCLC, small cell lung cancer; CEA, carcinoembryonic antigen; CYFRA 21-1, cytokeratin fragment 21-1; SCC, squamous cell carcinoma–associated antigen; ProGRP, pro–gastrin-releasing peptide; NSE, neuron-speciﬁc enolase; *, statistically significant finding

**Table 3:** Sensitivity and Specificity of the Tumour Markers in Various Comparisons; Cut-off as Per PackAge Inserts

TM/ TM combination	Sensitivity	Specificity
A. LC (N=160) vs no LC (N=18)		
CEA	62.50%	94.40%
CYFRA 21-1	78.10%	100.00%
NSE	68.10%	44.40%
ProGRP	31.30%	94.40%
SCC	40.00%	88.90%
Any one of 5 TMs abnormal	97.50%	33.30%
CEA [OR] CYFRA 21-1 [OR] ProGRP abnormal	91.30%	88.90%
B. NSCLC (N=147) vs SCLC (N=13)		
CEA	61.90%	30.80%
CYFRA 21-1	78.90%	30.80%
SCC	42.90%	92.30%
NSE1	76.90%	32.70%
ProGRP1	92.30%	74.10%

TM, tumour marker; LC, lung cancer; NSCLC, non-small cell lung cancer; SCLC, small cell lung cancer; CEA, carcinoembryonic antigen; CYFRA 21-1, cytokeratin fragment 21-1; SCC, squamous cell carcinoma–associated antigen; ProGRP, pro–gastrin-releasing peptide; NSE, neuron-speciﬁc enolase; 1this comparison was SCLC vs NSCLC.

**Table 4:** Study-Specific Cut-Offs Derived from Tumour Marker Levels Identified in the Present Study, and Cut-Off as Per Package Inserts

Tumor Marker	Unit	Package insert cut-offs	Study-specific cut-offs
CEA	ng/ml	4.7	2.23
CYFRA	ng/ml	3.3	2.91
NSE	ng/ml	16.3	20.64
ProGRP	pg/ml	68.3	50.15
SCC	ng/ml	2.6	2.25

CEA, carcinoembryonic antigen; CYFRA 21-1, cytokeratin fragment 21-1; SCC, squamous cell carcinoma–associated antigen; ProGRP, pro–gastrin-releasing peptide; NSE, neuron-speciﬁc enolase.

## Discussion

The main findings of this study are that it is possible to use the serum levels of 5 TMs – CEA, CYFRA 21-1, NSE, ProGRP, and SCC, to reduce the uncertainty of the diagnosis of LC and also, to support in the histological differentiation between NSCLC and SCLC. Measurement of serum TM levels represents a non-invasive, reliable, sensitive, and specific diagnostic modality. Interpretation of TM levels does not require specialised training and thus can be accomplished at the level of the PCP, and patients with suspicious pattern of TM levels can be promptly referred to higher centres for further work up and management, thereby enabling early diagnosis and prompt initiation of treatment. The combination of TMs was seen to have higher sensitivity for detecting LC than individual TMs, as also reported previously (Molina et al., 2016). A 2013 study from China found that the combination of CEA, CYFRA 21-1, and NSE to be the optimal combination for LC diagnosis, with a sensitivity of 75.76% and specificity of 88.57%; it is noteworthy that this study did not evaluate ProGRP, either alone or in combination (Wang et al., 2013). In fact, the 2018 Chinese guidelines for diagnosis and treatment of primary LC have included TMs as an essential component for diagnosis of LC, and also to predict the histological type of the LC (2019). We explored the potential of two different combinations of TMs, and the results indicate that the combination of all 5 TMs, and the combination of CEA, CYFRA 21-1, and ProGRP, by virtue of having high values of sensitivity and specificity can work as viable supplements to the existing diagnostic modalities for LC diagnosis in primary and secondary health care centres in India. 

These findings are significant especially from the Indian viewpoint. It is common knowledge that the high prevalence of tuberculosis in India often leads to misdiagnosis of LC patients as tuberculosis. A 2016 study from India reported that only 44.9% of all patients presenting to non-oncologists with respiratory symptoms were correctly diagnosed as having LC; out of the remaining misdiagnoses, the most frequent was tuberculosis at 17.8%, followed by lower respiratory tract infections at 5.6% (Ramachandran et al., 2016). Such cases of misdiagnoses of LC as tuberculosis is reported from various countries, especially the LMICs (low to middle income countries) (Singh et al., 2009; Hannan, 2016; Masamba et al., 2016). One main reason for such an observation is the relatively higher prevalence of tuberculosis in these countries compared to that of LC, leading to a lower index of suspicion towards LC. In India, the estimated crude incidence rate of LC in 2020 was 5.3 per 100,000, with 72,510 new cases, (WHO-IARC, 2020b) whereas the incidence of tuberculosis in 2019 was 193 per 100,000, with 2.64 million new cases.(WHO, 2020) Thus, the tendency of a PCP in the peripheral centres will be to evaluate for tuberculosis in patients who present with overlapping respiratory symptoms using modalities such as chest radiology and sputum examination. In case of unconfirmed diagnosis, the tendency is often to initiate anti-tuberculosis treatment keeping in mind the high prevalence of tuberculosis. The problems arising out of such a misdiagnosis, including such as loss of time, delayed referral, progression of LC to a higher stage, and the resulting mortality and declines in quality of life, have been highlighted repeatedly in the past, with the authors calling out for novel modalities for early diagnosis of LC. 

Treatment outcomes of most cancers are generally better with early treatment initiation, and early cancer diagnosis is imperative for the same. While population-based screening recommendations are well established for cancers of breast and cervix, such recommendations are lacking for LC, whose mortality rate is comparatively higher (Shankar et al., 2019).Methods such as sputum cytology and chest radiography have been tried for screening LC, and low-dose computer tomography (LDCT) is considered to be a reasonable tool for LC screening. However, concerns surrounding cost, access, and morbidity have been raised with respect to routine use of LDCT for lung cancer screening (Shankar et al., 2019). LDCT is also associated with other unwanted outcomes, including high false positive rates due to benign intrapulmonary lymph nodes or non-calcified granulomas, radiation induced cancer in the long term, and cancer overdiagnosis (Aberle et al., 2011).

Our research suggests that TM level estimation can be a viable solution to this diagnostic dilemma. Serum TM level evaluation can help in both lung cancer diagnosis in the periphery, and complement histological diagnosis of LC in specialist centres. Unlike LDCT, TM level estimation is not associated with unnecessary radiation risk, and does not require special training for interpretation. With an increase in the community acceptance of TMs, an increase in the demand for these TMs is also expected to go up; this would improve their accessibility. By the present study, we have also established the sensitivity and specificity of using all 5 TMs in combination, and a combination of three specific TMs (CEA, CYFRA 21-1, and ProGRP). While the combination of all 5 TMs had a higher sensitivity, the three-TM combination had improved specificity but at the cost of lower sensitivity. This suggests that using 5 TMs in combination offers the best modality to identify patients with possible LCs, by virtue of high sensitivity of the combination; using 3 TMs is also an alternative but offers a lower sensitivity than the former. However, considering the low number of non-LC subjects in our study, studies with larger sample size are required to confirm our findings. Including TM level estimation into the diagnostic armamentarium also solves some other frequently observed problems associated with histological LC diagnosis, such as inadequate tissue, non-availability of specific immune staining, doubtful histological LC diagnosis, and LC cases with mixed histological features. Thus, TM level estimation can be of potential value to chest physicians,PCPs, and histopathologists, on one hand, and to the patient and community at large on the other, in the diagnosis and management of LC.

TMs have come to occupy a prominent place in the management of various cancers, such as cancers of the colorectum, ovary, prostate, breast, and liver among others, wherein they help in the early diagnosis, and predicting prognosis, monitoring therapy and risk of recurrence (Sharma, 2009; Bagde et al., 2020). The acceptance of TMs in cancer management is gradually increasing to cancers of other organs, and LC is not an exception. While the use of single TMs is often not effective to screen LC, using multiple TMs can help in early diagnosis of LC. As mentioned earlier, combination of the 5 TMs evaluated in the present study have already been included in the Chinese lung cancer diagnosis guidelines (2019). The use of multiple TMs can prove to be cost-effective by virtue of aiding early diagnosis and initiating early treatment, and the associated reduction in mortality and morbidity, leading to socio-economic benefits. Studies in the past have suggested that while a 3-TM estimation is optimal, 2-TM estimation is cost-effective (Wang et al., 2013; Ma et al., 2015). In our study, we have established that the combination of CEA, CYFRA 21-1, and ProGRP is associated with higher rates of sensitivity and specificity. The cost-effectiveness of using TMs in different combinations for LC diagnosis in the Indian setting remains to be confirmed through future studies.

We observed that the sensitivity and specificity of both the TM combinations evaluated in our study could be further enhanced by adjusting the cut-offs to those derived from the ROC curves constructed from the study data. This hints at the possibility of population specific cut-offs leading to improvement in the accuracy of assessment. However, due to the selected sample limitation of our study the cut-off established in this study need to be confirmed by studies involving larger sample size, especially a larger number of non-cancer controls.

Our study needs to be interpreted in the light of a few limitations. We were unable to reach our targeted sample size because of the sudden onset of the COVID-19 pandemic. Being a tertiary care cancer centre, we often get referred cases from other centres. Thus, we were forced to include healthy subjects in the control group, and despite this our control group turned out to be disproportionately small. The small sample size in our study has impacted the power of the study, and the small number of controls also meant that a valid specificity analysis was not possible. We were also unable to obtain complete imaging data for the no LC group, hence, establishing the additional diagnostic value of adding TM assessment to clinical assessment for the LC diagnosis was not possible, as it has been done in a previous study by Molina et al.(Molina et al., 2016) Because of low numbers of patients with SCLC, it was not possible for us to enhance the excellent univariate performance of using ProGRP alone by adding further TMs to categorise SCLC patients from NSCLC patients among LC patients. Finally, the mean nodule size in our group of patients is large than that in other studies, because of the same reason. 

In conclusion, we have demonstrated the potential of combination of TMs to identify lung cancer cases from that of non-lung cancer. Testing of TMs is easy, non-invasive, does not require technical expertise, easy to interpret, and could lead the physician to suspect lung cancer early. This would ultimately lead to refer the patient in a timely manner to a specialist center especially in the light of overlapping pulmonary symptoms. Thus, TMs are of great value to general physicians and/or chest physicians practicing in non-specialist centres, especially in LMICs such as India where the prevalence of tuberculosis is high and patients with lung cancer are often misdiagnosed as having tuberculosis leading to poor treatment outcomes. TM evaluation also has a role to complement histological diagnosis of lung cancer. Further studies with larger sample size are warranted to confirm the findings of our study, and also to ascertain the cost-effectiveness of TM assessment specific to the Indian population. The additional value added by the TMs to the clinical assessment also needs to be explored with respect to the Indian setting.

## Author Contribution Statement

Anurag Mehta: Conceptualization; Data collection; Investigation; Methodology; Supervision; critical review of the manuscript. Anuj Parkash: Data collection; Investigation; Project administration; Supervision; critical review of the manuscript. Meenu Bhatia: Data collection; Investigation; critical review of the manuscript. All authors: Approval of the final version of the manuscript.

## Data Availability

All datasets leading to the results of the study are available with the corresponding author on reasonable request.
